# Supplementary material for: Refinement of a published gene-physical activity interaction impacting HDL-cholesterol: role of sex and lipoprotein subfractions
Source: medRxiv. 2024 Jan 24:2024.01.23.24301689. Preprint. [Version 1] doi: 10.1101/2024.01.23.24301689 (PMC10836120; doi:10.1101/2024.01.23.24301689)
Supplement: 2 [file NIHPP2024.01.23.24301689v1-supplement-2.pdf]

medRxiv preprint doi: https://doi.org/10.1101/2024.01.23.24301689; this version posted January 24, 2024. The copyright holder for this preprint (which was not certified by peer review) is the author/funder, who has granted medRxiv a license to display the preprint in perpetuity.
It is made available under a CC-BY 4.0 International license .

Supplementary Figures

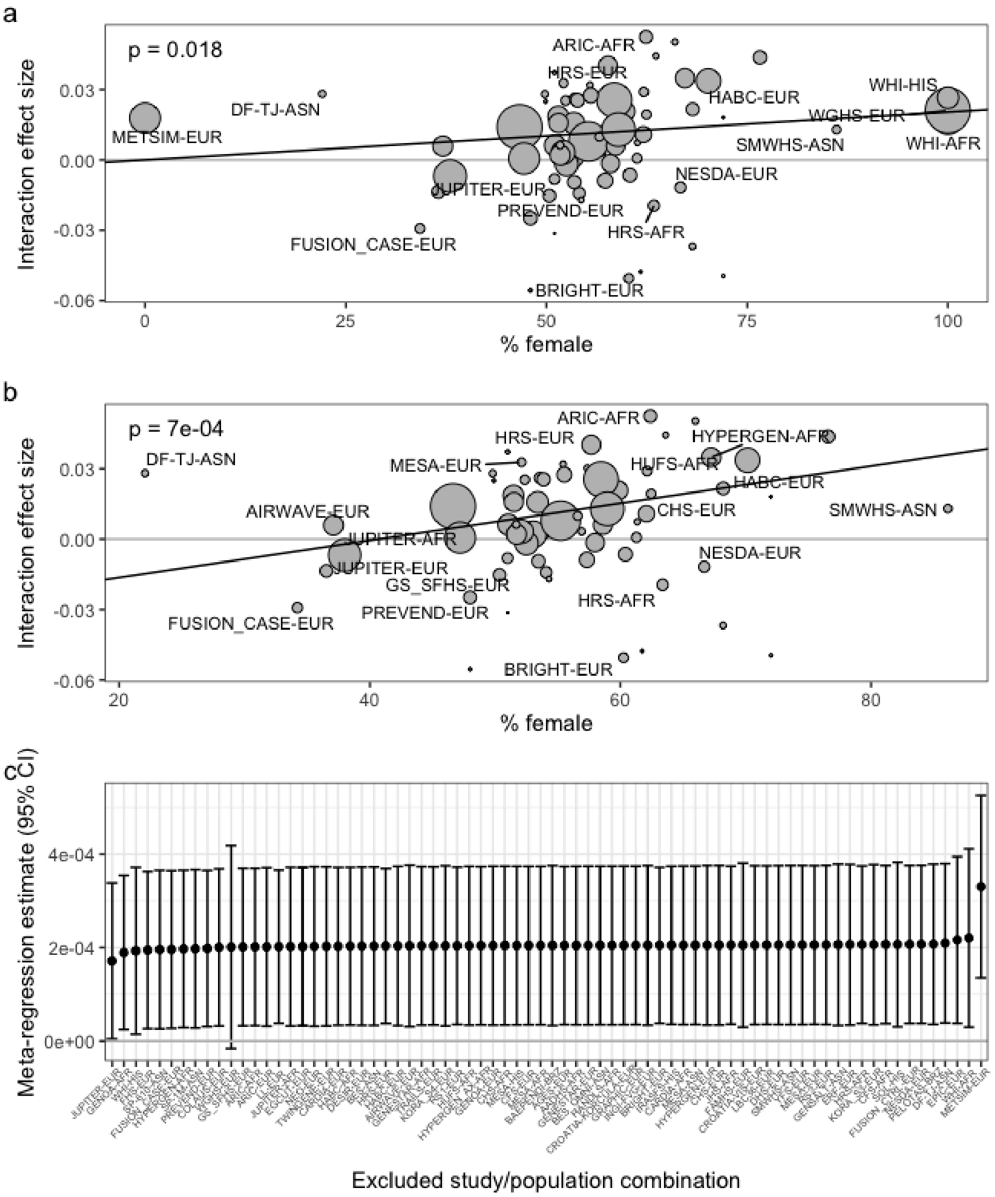

Supplementary Figure S1: Bubble plot describing the meta-regression of interaction summary statistics from Kilpeläinen et al. a) Interaction effect sizes from each cohort are plotted against the corresponding sex proportions for the primary meta-regression. Bubble size is proportional to the inverse variance of the interaction effect estimate. Study labels are shown for studies with N > 1,000. b) As in (a), but excluding all single-sex studies. c) Leave-one-out sensitivity analysis plots show meta-regression estimates and confidence intervals after excluding each study-population combination.

medRxiv preprint doi: https://doi.org/10.1101/2024.01.23.24301689; this version posted January 24, 2024. The copyright holder for this preprint (which was not certified by peer review) is the author/funder, who has granted medRxiv a license to display the preprint in perpetuity.
It is made available under a CC-BY 4.0 International license .

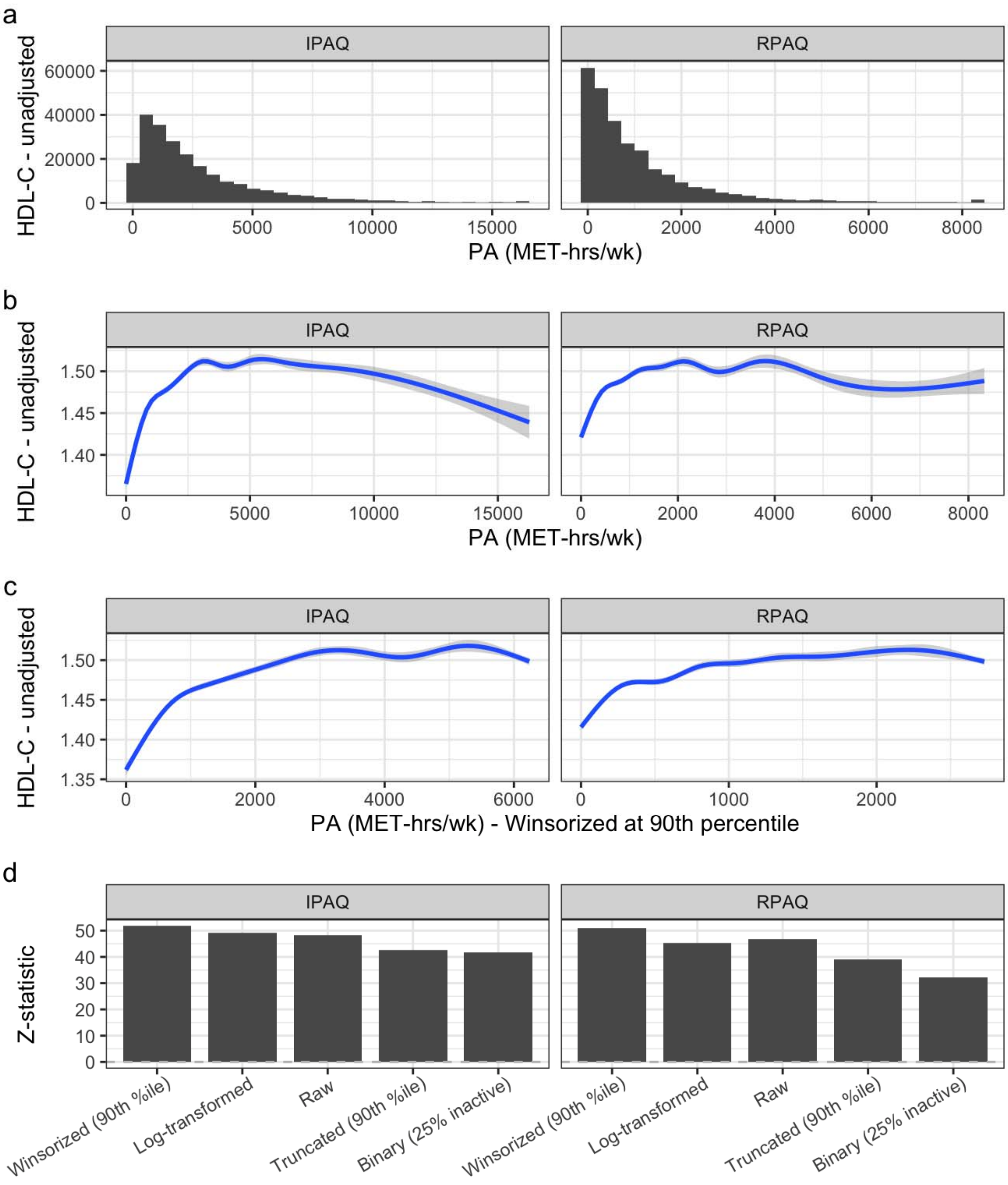

Supplementary Figure S2: PA-HDL-C main effects in UKB. a) Histogram of PA measured using questions based on the IPAQ (left) or RPAQ (right) questionnaires. b) Shrunken cubic spline fits of the relationship between PA and unadjusted mean HDL-C, with shadows corresponding to 95% CIs. c) As in (b), but using PA distributions winsorized at the 90th percentile. d) Adjusted main effect z-statistics according to PA transformation.

medRxiv preprint doi: https://doi.org/10.1101/2024.01.23.24301689; this version posted January 24, 2024. The copyright holder for this preprint (which was not certified by peer review) is the author/funder, who has granted medRxiv a license to display the preprint in perpetuity.
It is made available under a CC-BY 4.0 International license .

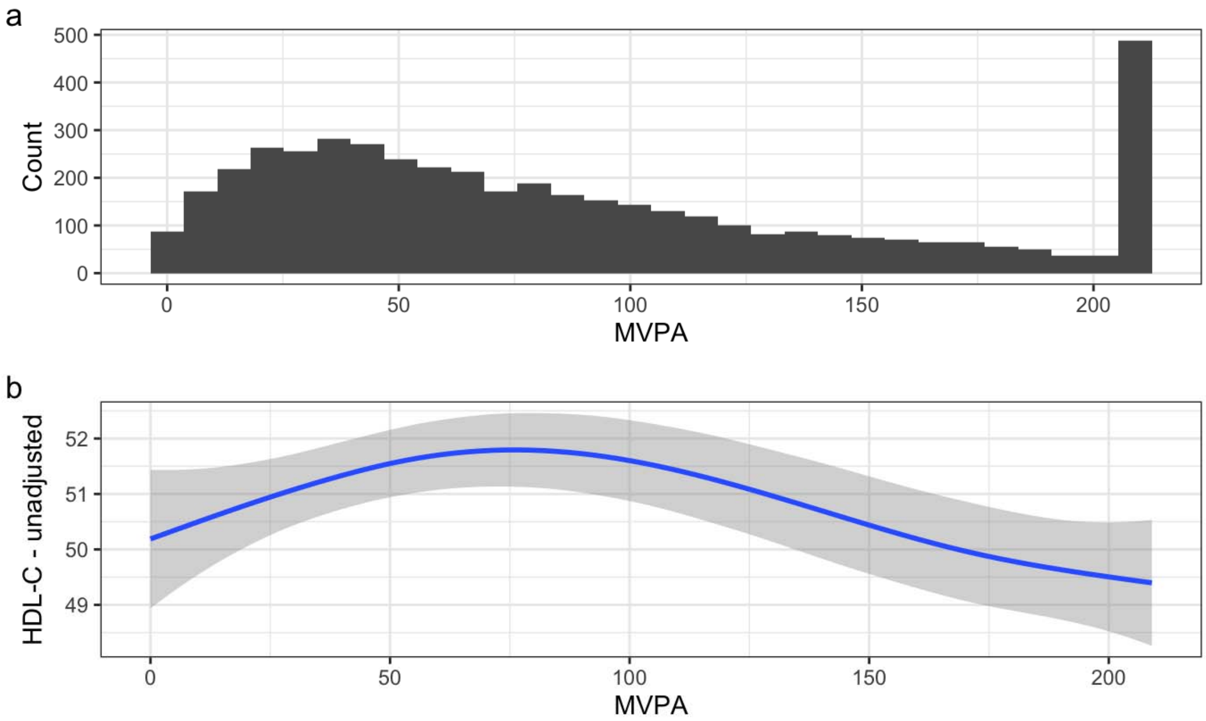

Supplementary Figure S3: PA-HDL-C main effects in MESA. a) Histogram of questionnaire-based moderate-to-vigorous PA estimates. b) Shrunken cubic spline fit of the relationship between PA and unadjusted mean HDL-C, with shadows corresponding to 95% CIs.

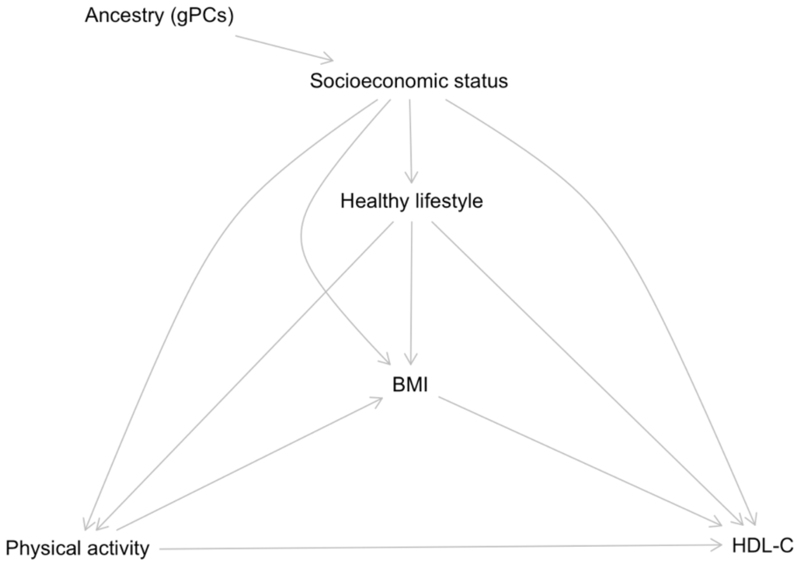

Supplementary Figure S4: Directed acyclic graph informing covariate selection based on PA-HDL-C main effect.
